# Input of terrestrial organic matter linked to deglaciation increased mercury transport to the Svalbard fjords

**DOI:** 10.1038/s41598-020-60261-6

**Published:** 2020-02-26

**Authors:** Haryun Kim, Sae Yun Kwon, Kitack Lee, Dhongil Lim, Seunghee Han, Tae-Wook Kim, Young Ji Joo, Jaesoo Lim, Moo-Hee Kang, Seung-Il Nam

**Affiliations:** 1 Fundamental Research Division, National Marine Biodiversity Institute of Korea, 33662 Janghang, South Korea; 2 Division of Environmental Science and Engineering, Pohang University of Science and Technology, 37673 Pohang, South Korea; 3 South Sea Research Institute, Korea Institute of Ocean Science and Technology, 53201 Geoje, South Korea; 4 School of Earth Sciences and Environmental Engineering, Gwangju Institute of Science and Technology (GIST), 61005 Gwangju, South Korea; 5 Division of Environmental Science and Ecological Engineering, Korea University, 02841 Seoul, South Korea; 6 Division of Polar Paleoenvironment, Korea Polar Research Institute, 21990 Incheon, South Korea; 7 Geological Research Division, Korea Institute of Geosciences and Mineral Resources, 34132 Daejeon, South Korea; 8 Petroleum and Marine Research Division, Korea Institute of Geoscience and Mineral Resources, 34132 Daejeon, South Korea

**Keywords:** Environmental impact, Marine chemistry

## Abstract

Deglaciation has accelerated the transport of minerals as well as modern and ancient organic matter from land to fjord sediments in Spitsbergen, Svalbard, in the European Arctic Ocean. Consequently, such sediments may contain significant levels of total mercury (THg) bound to terrestrial organic matter. The present study compared THg contents in surface sediments from three fjord settings in Spitsbergen: Hornsund in the southern Spitsbergen, which has high annual volume of loss glacier and receives sediment from multiple tidewater glaciers, Dicksonfjorden in the central Spitsbergen, which receives sediment from glacifluvial rivers, and Wijdefjorden in the northern Spitsbergen, which receive sediments from a mixture of tidewater glaciers and glacifluvial rivers. Our results showed that the THg (52 ± 15 ng g^−1^) bound to organic matter (OM) was the highest in the Hornsund surface sediments, where the glacier loss (0.44 km^3^ yr^−1^) and organic carbon accumulation rates (9.3 ~ 49.4 g m^−2^ yr^−1^) were elevated compared to other fjords. Furthermore, the δ^13^C (–27 ~ –24‰) and δ^34^S values (–10 ~ 15‰) of OM indicated that most of OM were originated from terrestrial sources. Thus, the temperature-driven glacial melting could release more OM originating from the meltwater or terrestrial materials, which are available for THg binding in the European Arctic fjord ecosystems.

## Introduction

Increasing temporal trends of total mercury (THg) concentration in the sediments of the Arctic continental shelves and lakes have recently been reported^1,2^. These increasing levels have been proposed to be caused by the long-distance transport of anthropogenic mercury (Hg) from industrialized countries to the Arctic, given its long atmospheric lifetime of 0.3 to 2 years^3,4^. In addition, it has been shown that ‘Atmospheric Mercury Depletion Events’ (AMDE) in the spring can lead to high Hg deposition in the Arctic Ocean. Reactive Hg (Hg^2+^) is rapidly formed through *in situ* oxidation of gaseous Hg° by halogens (i.e., atomic Br and radical BrO), which are generated by solar irradiation through O_3_ destruction^5–7^. However, previous measurements of atmospheric Hg deposition in the Arctic regions have revealed relatively low deposition rates of <5 µg m^−2^ yr^−1^ ^8^. Recent studies have suggested that natural processes governing Hg release via riverine transport^9^, soil runoff, and/or discharge of water coming from thawing permafrost^10^ could be important sources of Hg to the Arctic environments.

Fjords on Svalbard, the main island of the Svalbard archipelago, is among the most studied fjord systems in the world^11^. Previous studies have focused on the effects of local and/or long-range transport, oceanic currents, and long-term sediment deposition rates (i.e., geological and chemical processes that control Hg distribution in surface and core sediments)^4,12–17^ on spatial distribution and the extent of accumulation of Hg in the fjord sediment. Recent studies have shown that fjords are effective sequesters of organic carbon among other marine environments^18,19^. Given that Hg demonstrates a strong binding affinity towards organic matter (OM), we investigated the influence of OM on the spatial distribution of THg in the fjord systems. To address this research, we collected 35 surface sediment samples from three fjords in Spitsbergen that are affected by various glacial influences: Wijdefjorden in the northern Spitsbergen,  Dicksonfjorden in the central Spitsbergen and Hornsund in the southern Spitsbergen. Each study area is characterized by distinct glacier dynamics, bedrock lithology, and oceanic currents with meltwaters. The THg, total organic carbon (TOC), total nitrogen (TN), and total sulfur (TS) contents, and their isotope values were analyzed in the surface sediments. Six trace metals (As, Cu, Ni, Zn, Cr, and Pb) and twelve proxy tracers, which indicate the influence of weathering and redox processes, were also analyzed to examine the potential controlling factors for the spatial distribution of THg in fjord surface sediments. Our study examines the sources and factors that influence the deposition (i.e., sediment composition, runoff, weathering, and anthropogenic influence), and the spatial distribution of Hg in fjord sediments in the Svalbard archipelago.

## Results

### Geological and glacier properties, and organic carbon (OC) accumulation rates

The bedrock mainly represents Paleozoic strata in Wijdefjorden and Dicksonfjorden, and Mesozoic strata in Hornsund (Table 1). Therefore, the bedrock components in Hornsund are relatively younger compared to other fjords. The glacier-fed systems were marine-terminating types for Wijdefjorden and Hornsund, whereas Dicksonfjorden had a land-terminating glacier-fed system. The total glacier area, the annual loss glacier volume, and the glacier retreat rate were significantly higher in Hornsund than Wijdefjorden and Dicksonfjorden (Table 1). Furthermore, sedimentation and OC accumulation rates were higher in Hornsund compared to other fjords (Table 1)^20–24^. However, sedimentation rates can exceed several tens of cm yr^−1^ in the vicinity of terrestrial sources^25,26^.

**Table 1:** Physical, geological^52^ and glacier^21,30,31,62–65^ properties and surface organic carbon (OC) accumulation rates of the Wijdefjorden, Dicksonfjorden and Hornsund surface sediments in the Svalbard archipelago.

Characteristics	Wijdefjorden	Dicksonfjorden	Hornsund
Latitude (°N)	79.0~80.3	78.4~78.8	69.3~77.1
Longitude (°E)	15.4~16.2	15.3~15.4	15.8~16.7
Range of water depth (m)	112~322	37~109	37~193
Bedrock Lithology^66^	Phyllite sandstone	Sandstone, black mudstone & calcareous shale	Phyllite, Mesozoic shales & sandstones
Age^66^	Meso/Paleozoic & Devonian	Carboniferous/ Permian & Devonian	Neoproterozoic & Jurassic/Cretaceous
Glacier fed-system	Marine terminating	Land terminating	Marine terminating
Summer temperature (°C)	−3~−1	0~1	2~3
Catchment area (km^2^)	1,100^21^	1,013^67^	>1,200^30^
Total glacier area (km^2^)	500	283	802^*^
Loss balance of glacier mass^¥^ (w. eq. m yr^−1^)	−0.26	−0.26	−0.55
Annual loss glacier volume (km^3^ yr^−1^)	0.13	0.07	0.44
Glacier retreat rate (m yr^−1^)	<10	35	70
Glacier retreat area rate (km^2^ yr^−1^)	—	0.24	1.6
Sedimentation rate (cm yr^−1^)	0.01^20,68^	0.1~0.4^69^	0.17~0.66^22^
Mass accumulation rate in sediment^§^ (g m^−2^ yr^−1^)	40	1100	1800
OC accumulation rate in sediment^£^ (g m^−2^ yr^−1^)	0.3~0.8	1.3~11.3	9.3~49.4

*Area of tidewater glacier is 781 km^2^.

^¥^These values were estimated using a geodetic method comparing the glacier thickness over years in the maps of a glacier made at two different points in time.

^§^Mass accumulation rate (MAR) was calculated using the following equation:

MAR (g cm^−2^ yr^−1^) = Sedimentation rate (cm yr^−1^) × [1-Porosity] × Bulk density (g cm^−3^)

We used 0.7 and 1.85 (g cm^−3^) for porosity^18^ and bulk density^18,20^, respectively.

^£^OC accumulation rate was calculated by multiplying OC by MAR.

### THg and other trace metal concentrations

**Figure 1:**
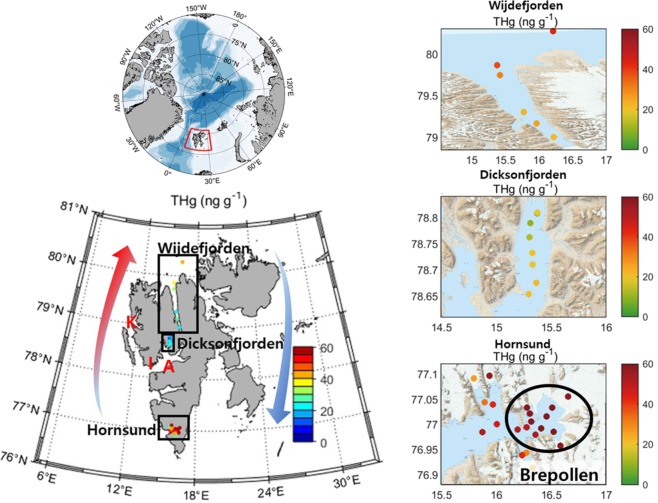
Spatial distributions of absolute THg concentration in the surface sediment of the Wijdefjorden, Dicksonfjorden, and Hornsund of Svalbard archipelago. The K, I, and A mean Kongsfjorden, Isfjorden, and Adventfjord, respectively. Red and blue arrows indicate warm Atlantic and cold Arctic currents, respectively. The map was created using an S100 Raster Data Set^70^ in Matlab 2018a version^71^.

The THg concentrations in the surface sediments were the highest in Hornsund (52 ± 15 ng g^−1^, *n* = 21), followed by Wijdefjorden (30 ± 9 ng g^−1^, *n* = 6), and Dicksonfjorden (16 ± 6 ng g^−1^, *n* = 8) (Table 2). The average THg concentration in Hornsund surface sediment was three times higher than in Dicksonfjorden. The highest THg concentrations occurred in Brepollen, a bay surrounded by retreating tidewater glaciers in the innermost parts of Hornsund (Fig. 1 and Table S1). The THg mass accumulation rate in Hornsund (92 ± 27 μg m^−2^ yr^−1^) was higher than those of Wijdefjorden (1.2 ± 0.4 μg m^−2^ yr^−1^) and Dicksonfjorden (17 ± 6 μg m^−2^ yr^−1^) in Svalbard. The THg mass accumulation rate in Hornsund was comparable to that of the Huang He River (100 ± 15 μg m^−2^ yr^−1^), which is well known for high sedimentary Hg accumulation rates due to industrial and riverine input, but much higher compared to East China (36 ± 2 μg m^−2^ yr^−1^) and Yellow seas (29 ± 2 μg m^−2^ yr^−1^) (Table 3).

**Table 2:** Averages or range of chemical properties of surface sediments from Wijdefjorden, Dicksonfjorden, and Hornsund in the Svalbard archipelago.

	THg in sediments (ng g^−1^)	THg in rock^52^ (ng g^-1^)	TOC (%)	TN (%)	TS (%)	TOC/TN ratio	δ^13^Corg (‰)	δ^15^N (‰)	δ^34^S (‰)
Wijdefjorden	30 ± 9	0~0.89	1.20 ± 0.54	0.14 ± 0.10	0.15 ± 0.06	8.5 ± 0.5	−23~−22	4.7~5.3	8~15
Dicksonfjorden	16 ± 6	5~13	0.59 ± 0.33	0.07 ± 0.03	0.09 ± 0.04	7.8 ± 2.4	−25~−24	2.7~5.4	14~17
Hornsund	52 ± 15	0~0.89	1.68 ± 0.55	0.11 ± 0.03	0.25 ± 0.10	14.5 ± 2.8	−27~−24	2.0~5.9	−10~15

**Table 3:** THg concentrations and THg mass accumulation rates in surface sediments from various sites.

Sites^*^	Sample No.	THg (ng g^−1^)	MAR (g cm^−2^ yr^−1^)	THg MAR (μg m^−2^ yr^−1^)
Huang He River	22	20 ± 3	0.5	100 ± 15
Changjiang River	24	105 ± 11	0.87	914 ± 96
East China Sea	27	18 ± 1	0.2	36 ± 2
Yellow Sea	195	19 ± 1	0.15	29 ± 2
East Sea	6	89 ± 22	0.02	18 ± 4
Okhotsk Sea	27	91 ± 30	0.02	18 ± 6
Bering Sea	11	49 ± 9	0.07	34 ± 6
Western Arctic Ocean	7	78 ± 8	0.04	31 ± 3
Alaska permafrost^37^	588	43 ± 30	—	—
Wijdefjorden	6	30 ± 9	0.004	1.2 ± 0.4
Dicksonfjorden	8	18 ± 7	0.11	17 ± 6
Hornsund	21	52 ± 15	0.18	92 ± 27

*The data from Huang He River, Changjiang River, East China Sea, East Sea, Okhotsk Sea, Bering Sea and western Arctic Ocean were referred from Kim *et al*.^38^.

**Figure 2:**
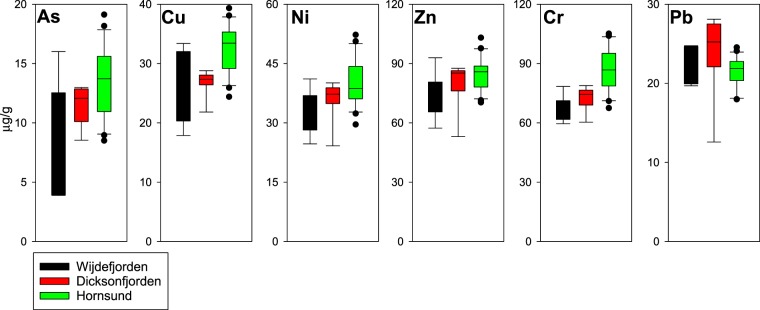
Box plots of trace metal concentrations (As, Cu, Ni, Zn, Cr, and Pb) in the surface sediments of the Svalbard fjords that were studied.

The THg concentration in Wijdefjorden showed a decreasing trend from terrestrial to coastal (offshore) regions. The THg concentrations in Dicksonfjorden were relatively constant throughout the study area (Fig. 1). Other trace metal concentrations, including As, Cu, Ni, Zn and Cr were higher in Hornsund than in Wijdefjorden and Dicksonfjorden; however, Pb concentrations were not significantly different among the sites (Fig. 2).

### Correlation between THg, TOC and TS concentrations

The concentrations of TOC (1.68 ± 0.55%, *n* = 21) and TS (0.25 ± 0.10%, *n* = 20) and TOC/TN ratios (14.5 ± 2.8, *n* = 21) were the highest in Hornsund, followed by Wijdefjorden (TOC; 1.2 ± 0.54%, *n* = 6, TS; 0.15 ± 0.06, *n* = 3, TOC/TN ratio 8.5 ± 0.5, *n* = 6), and Dicksonfjorden (TOC; 0.59 ± 0.33%, *n* = 8, TS; 0.09 ± 0.04%, *n* = 6, TOC/TN ratio 7.4 ± 2.4, *n* = 8) (Table 2 and S1). The average δ^13^C_org_ value was the highest in Wijdefjorden (ranging from −23 to −22‰, average of −22.8 ± 0.4‰, *n* = 6), followed by Dicksonfjorden (ranging from −25 to −24‰, average of −24.5 ± 0.5‰, *n* = 8), and Hornsund (ranging from −27 to −24‰, average of −25.6 ± 0.7‰, *n* = 21) (Table 2 and S1). The average δ^34^S value was notably lower in Hornsund (ranging from −10 to 15‰, average of 1.3 ± 8.2‰, *n* = 20) compared to other sites (Wijdefjorden: ranging from 8 to 15‰, average of 10.9 ± 3.8‰, *n* = 3; and Dicksonfjorden, ranging from 14 to 17‰, average of 15.6 ± 1.2, *n* = 6) (Tables 2 and S1).

**Figure 3:**
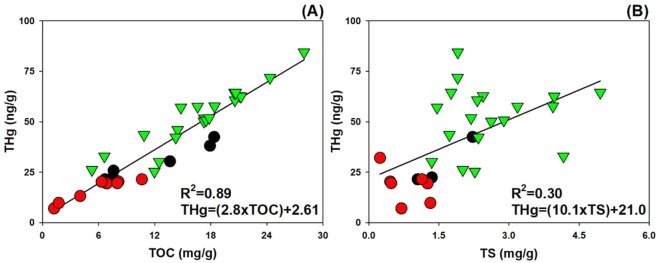
Relationships between TOC (**A**), TS (**B**) and THg concentrations of the surface sediments in the Svalbard fjords that were studied.

**Figure 4:**
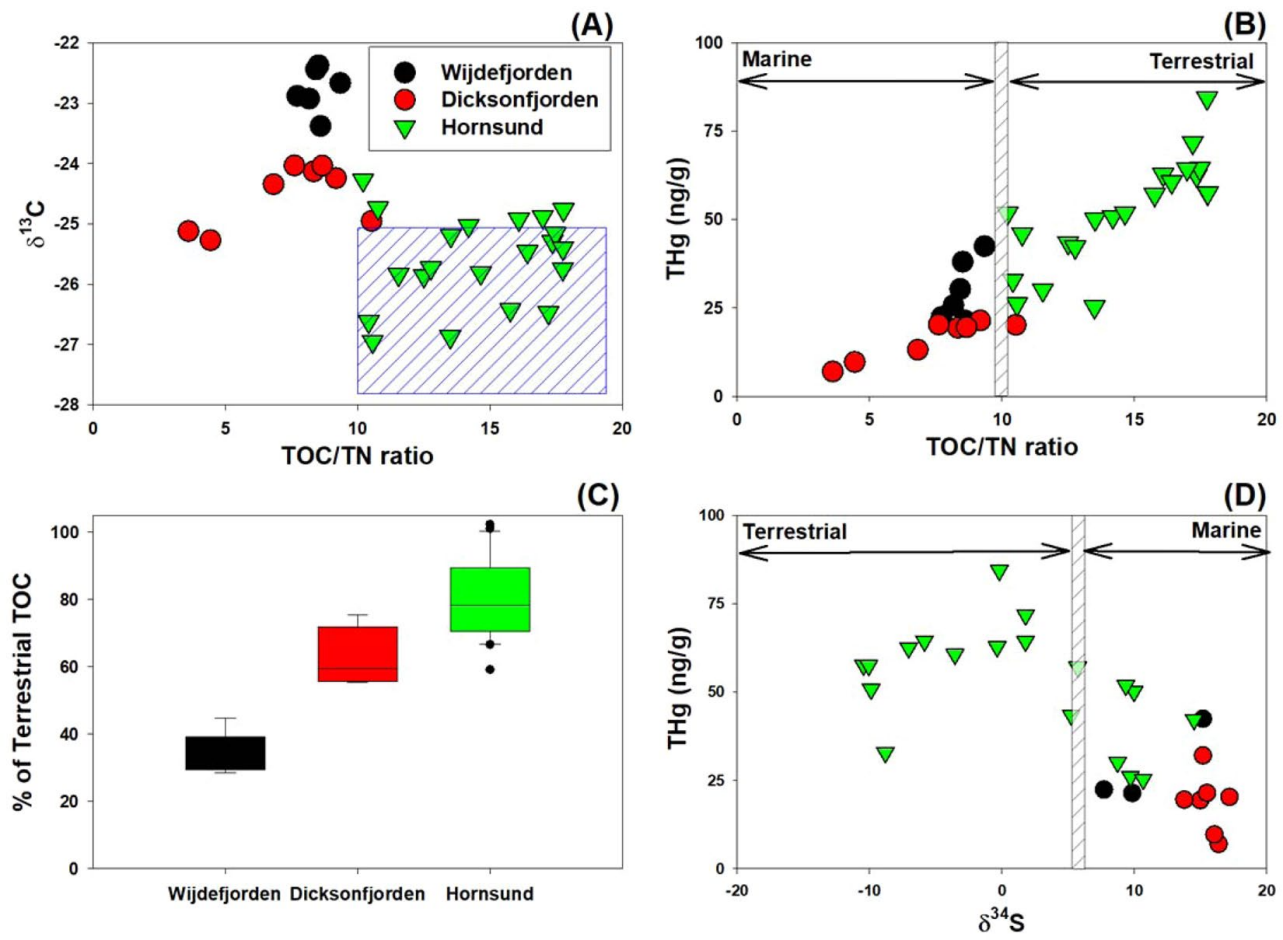
Relationship between TOC/TN ratio and δ^13^C values (‰) (**A**), THg and TOC/TN ratios (**B**), the percentage of terrestrial organic carbon (*F*_*terr*_; **C**), and THg and δ^34^S (‰) values (**D**) in the surface sediments in the Svalbard fjords studied. The blue box (**A**) indicates the range of TOC/TN ratio and δ^13^C (‰) that are derived from terrestrial OM.

In all fjord systems studied, the concentrations of TOC (Fig. 3A) and TS (Fig. 3B) were strongly correlated with THg concentrations. The positive relationships between TOC and THg remained valid after THg and TOC values of individual sediments ([THg]_sample_ and [TOC]_sample_) were normalized to mean fjord values ([THg]_Fjord mean_ and [TOC]_Fjord mean_) (Fig. S1). The TOC/TN ratios also correlated well with THg concentration (Fig. 4B). Interestingly, the THg in Hornsund had a positive relationship with the TOC/TN ratio, ranging from 10 to 20. Wijdefjorden and Dicksonfjorden also showed positive correlations between THg concentrations and TOC/TN ratios with ratios less than 10 (Fig. 4B).

### Relative percentage of terrestrially-derived TOC

The Hornsund sediments had relatively negative δ^13^C (‰) values similar to the endmembers for terrestrially-derived OM, while the Wijdefjorden and Dicksonfjorden had higher δ^13^C (‰) values, characterized by marine-derived OM (Fig. 4A–C). Using the δ^13^C (‰) endmembers for terrestrial and marine sources (see Eqs. 1 and 2 in the Methods section), the Hornsund sediment was composed of a relatively high proportion of terrestrial OM (*F*_*terr*_; an average of 78 ± 17% terrestrial source) (Fig. 4C). The relative contribution of terrestrial OM to surface sediments (*F*_*terr*_) decreased with increasing latitude, suggesting that TOC in the Wijdefjorden sediments was predominantly composed of marine OM (with an average of 35% terrestrial sources and 65% marine sources) (Fig. 4C). In addition, 14 out of 20 sites in the Hornsund studied showed depleted δ^34^S (‰) values, which were within the endmember of terrestrially derived-OM (Fig. 4D).

### Relationships between detrital/redox proxy metals and THg concentrations

To investigate the effects of weathering (or erosion) on THg concentrations, THg was plotted with detrital and lithogenic proxy metals such as Al, Zr, Ti, Rb, Th and Hf, which are often used as indicators of these influences^27–29^. Our results showed positive relationships between the concentrations of THg, Zr, Ti and Hf across all sites (*p* < 0.05; Table 4 and S2). To investigate the effect of anaerobic reduction on THg concentrations, redox proxy metals including Fe, Mn, Cr, V, Mo and U, were plotted with THg concentrations. The results indicated positive correlations between THg, Cr and V concentrations in the surface sediments of the Svalbard fjords (Tables S3, p < 0.05). The discussion of redox influence on spatial THg distributions was described in the supplementary information in detail.

**Table 4:** Pearson correlation coefficients and *p* values between THg, TOC, and metals in surface sediment from Svalbard fjords.

Other trace metals							
		As	Cu	Ni	Zn	Cr	Pb
THg	R^2^	0.54	0.45	0.08	0.13	0.53	−0.04
	*P* value	**<0.01**	**0.01**	0.65	0.47	**<0.01**	**0.02**
TOC	R^2^	0.41	0.38	0.01	0.04	0.45	−0.34
	*P* value	**0.01**	**0.02**	0.96	0.82	**0.01**	0.05
**Detrital & Lithogenic weathering proxies**							
		Al	Zr	Ti	Rb	Th	Hf
THg	R^2^	0.28	0.41	0.41	‒ 0.22	‒ 0.29	0.56
	*P* value	0.11	**0.01**	**0.01**	0.21	0.09	**<0.01**
TOC	R^2^	0.26	0.52	0.46	‒0.21	‒0.26	0.61
	*P* value	0.13	**<0.01**	**0.01**	0.22	0.13	**<0.01**

## Discussion

Hornsund has one of the largest areas of glacier coverage in the catchment area and a broad calving front in the Svalbard fjord systems^30,31^. Since the 1990s, the Hornsund glaciers have retreated rapidly in Svalbard (~70 m yr^−1^ on average) and had a large annual volume of loss glaciers (0.44 km^3^ yr^−1^ on average) due to higher summer temperature (by 2~3 °C) compared to other fjords^30,31^. Therefore, Hornsund with tidewater glaciers is particularly susceptible to the influence of glacier melting, which releases fresh meltwater and drifting ice to fjords. These processes have shown to supply modern and ancient OM, trace metals, and nutrients both directly by releasing glacier-trap resources and indirectly by exposing terrestrial landscape prone to runoff to fjord ecosystems^32,33^. In fact, high sediment (0.17 ~ 0.66 cm yr^−1^) and organic carbon (OC) accumulation (9.3 ~ 49.4 g m^−2^ yr^−1^) rates were observed in Hornsund. The THg concentrations (52 ± 15 ng g^−1^) and THg mass accumulation rates (THg MAR) in Hornsund (94 ± 27 μg m^−2^ yr^−1^) were comparable to those of Huang He River (THg; 20 ± 15 ng g^−1^, THg MAR; 100 ± 15 μg m^−2^ yr^−1^) and higher than East China (THg; 18 ± 1 ng g^−1^, THg MAR; 36 ± 29 μg m^−2^ yr^−1^) and Yellow Seas (THg; 19 ± 1 ng g^−1^, THg MAR; 29 ± 2 μg m^−2^ yr^−1^), which are characterized by substantial coastal runoff and the largest local anthropogenic Hg emission in the world (Table 3). We speculate that the deglaciation is the main process leading to increased Hg release into the Svalbard fjord environment as supported by the elevated trace metal concentrations (As, Cu, Ni, Zn and Cr) and TOC in addition to THg in the Hornsund sediments.

The deglaciation-mediated Hg release to the Svalbard fjord environment is likely facilitated by OM. It has long been suggested that  OM mediates the transport of Hg from terrestrial to the adjacent aquatic environment. Negatively charged surfaces of OM are enriched with carboxyl functional groups (RCOO^−^), and thiolates (RS^−^) in OM behave as ligands to form a transition metal thiolate complex, which leads to a strong binding affinity with trace metals including Hg^34–36^. These associations among OM, thiolates, and THg contents can explain the positive relationships among them as observed in the Svalbard fjords (Fig. 3). These results support earlier studies showing that sediments with high OM are typically characterized by high Hg concentrations in the North Slope of Alaska, the Yukon River basin in the Canadian Arctic, and Kongsfjorden on Spitsbergen^10,16,37,38^.

The observed TOC/TN ratios and the δ^13^C values of the sediments suggest that Hg bound to terrestrial OM, which is either released directly via glacial meltwater or indirectly by exposing terrestrial landscape, acts as the primary Hg source to the Hornsund sediments in contrast to other locations of Wijdefjorden and Dicksonfjorden. Terrestrially-derived OM has a TOC/TN ratio larger than 10 and a δ^13^C value ranging from −29.3 to −25.5‰ in the Arctic, due to the influence of C_3_ plants and ancient OM^39–43^. The OM from marine sources displays a lower TOC/TN ratio (i.e., <8) and a heavier δ^13^C value (> −25‰)^42–46^. Our results suggest that OM in the Hornsund sediments, which display relatively high TOC/TN ratios and relatively depleted δ^13^C values, is mainly derived from modern and ancient terrestrial OM (Fig. 4A,B). The Hornsund sediments also had the highest THg concentration. The Wijdefjorden sediments consist mainly of OM originated from marine sources (Fig. 4A,B). The Dicksonfjorden sediments, which have average values for both TOC/TN ratio and δ^13^C, were probably generated by the mixing of both terrestrially- and marine-derived OM (Fig. 4A,B).

To quantify the contribution of terrestrially-derived OM to fjord sediments, we calculated *F*_*terr*_ (the percentage of terrestrial OM in the samples) using the δ^13^C (‰) end members derived from terrestrial and marine sources^39^. Due to the complex lithology of the Svalbard fjord sediments, which can lead to an extensive range of δ^13^C end members for terrestrial OM, we used measured δ^13^C end members from Spitsbergen (Hornsund and Adventfjord) for terrestrial sources, and sites west of Svalbard for marine sources (45 samples collected in the open ocean off Svalbard), and calculated a mixing model with C and N isotopes, and TOC/TN ratios^43,47,48^. The results indicated that the Hornsund sediments were composed of both modern and ancient terrestrial OM (78%), which is consistent with earlier studies^22,43,48^. The contribution of OM derived from marine sources was relatively higher in the Wijdefjorden (65%) and Dicksonfjorden (37%) sediments (Fig. 4C). This finding implies that the sediments in Hornsund are influenced by OM carried in meltwater from glaciers, which promotes the release of Hg bound to OM from terrestrial sources.

To support our hypotheses that OM in Hornsund mostly came from terrestrial sources, we further analyzed the isotopic values of sulfur (δ^34^S) (Fig. 4D). The δ^34^S values have been used as a tool for distinguishing terrestrial OM from marine-derived OM. The δ^34^S values of terrestrially- and marine-derived OM have larger differences compared to δ^13^C values, and the assimilation and degradation of OM lead to a smaller degree of δ^34^S fractionation (by 1 ~ 2‰)^49,50^. Thus, δ^34^S values can be a conservative tracer accounting for terrestrially- and marine-derived OM^51^. Generally, the terrestrially-derived OM has lower δ^34^S values (6.3‰) compared to marine-derived OM (18.1‰)^49,50^. In our results, Hornsund had notably lower δ^34^S isotopic values (ranging from −10 to 15‰, average of 1.3 ± 8.2‰, *n* = 20) than those of Wijdefjorden (ranging from 8 to 15‰, average of 10.9 ± 3.8‰, *n* = 3) and Dicksonfjorden (ranging from 14 to 17‰, average of 15.6 ± 1.2‰, *n* = 6), indicating that the surface sediment of Hornsund was mainly composed of terrestrial sources (Table 2 and Fig. 4D). In particular, lower δ^34^S values in the Hornsund were mainly observed in the inner part of fjord, Brepollen, which had high THg concentration and was significantly affected by tidewater glaciers (Fig. 1).

While terrestrially-derived OM is thought to be the main mediator for the transport of Hg to the Svalbard fjord sediments, the source of Hg still remains a question. Fjord sediments have been shown to receive Hg via three major sources-direct atmospheric deposition (in the form of precipitation), industrial runoff, and riverine input. The fjord sediments in Svalbard are atypical such that there are few major point sources of anthropogenic Hg. The recent records of atmospheric Hg deposition in the Arctic region (>5 ug m^−2^ yr^−1^) have also shown particularly low values compared to other regions of the world (East China and Yellow seas: 86 ~ 133 ug m^−2^ yr^−1^) but the THg concentration of the Svalbard fjord sediments are comparable to several remote regions (Table 3)^8,9,38^. Based on a number of analyses performed in this study, we speculate that the dominant Hg source to the Svalbard sediments is through weathering of bedrock. Although the THg concentrations in the bedrock were higher in Dicksonfjorden than Wijdefjorden and Hornsund (Table 2), Ottesen *et al*.^52^ reported that the Jurassic or Cretaceous and Triassic bedrock in Hornsund contain higher Hg concentrations (ranging from 40 to 80 ng g^−1^) compared to the Devonian rock in the Wijdefjorden and Dicksonfjorden (ranging from 5 to 30 ng g^−1^)^52^. To assess the contribution of weathering or erosion, we analyzed the detrital metals degraded from bedrocks (Al, Zr, Ti, Rb, Th and Hf), which are the most abundant components of minerals, less immobile in sediments and exclusively derived from natural sources (i.e., no anthropogenic source exists)^29,53^.Observed positive relationships between THg and some detrital proxies (Zr, Ti, Hf) suggest that weathering or erosion may have liberated Hg, which is then sequestered and transported by terrestrially-derived OM to the sediment (Table 3). Bełdowski *et al*.^15^ also report a noticeable impact of weathered bedrock minerals on sediment THg concentration that were transferred from glacial meltwater in Kongsfjorden as well as in Hornsund. It should also be noted that the rapid deglaciation event has likely facilitated the erosion of bedrock particularly in Hornsund, which explains the highest THg observed in that location. In summary, the lithogenic characteristic such as bedrock composition and degree of weathering or erosion could be one of the significant factors determining the spatial THg distribution in the sediment.

Given the absence of local or regional anthropogenic disturbances (which are described in the supplementary information in detail), another natural Hg source to the Svalbard fjord sediments is by sinking of particulate OM. A recent study^54^ has suggested that high Hg concentrations observed in lake sediments located near Ny-Ålesund are caused by increased phytoplankton biomass due to global warming. Phytoplankton carcasses can take up Hg from the water column and subsequently deposited Hg directly into the sediment^54^. Based on the relatively heavier δ^13^C values and lower TOC/TN ratios^39,43^, which are indicators of the influence of marine phytoplankton, it is possible that algal scavenging processes have influenced the Wijdefjorden and Dicksonfjorden sediments in recent periods. This process, however, does not explain the elevated sediment THg concentration in Hornsund, which is mainly consisted of terrestrial OM

In summary, Hornsund experiencing rapid glacial retreat rates and a wide calving front showed the highest concentrations of THg (52 ± 15 ng g^−1^) and TOC (1.68 ± 0.55%), compared to the Wijdefjorden and Dicksonfjorden fjords. Our study suggests that the sedimentary THg contents of all three fjords are mainly derived from weathered and deposited minerals, and both modern and ancient OM released from melting glaciers act as the primary transport mechanism for Hg. Thus, the large amount of THg released from the terrestrial to the fjord system is likely to affect Hg cycling in the water column as well as the sediments of the Svalbard fjords.

## Methods

### Site description and sampling

Spitsbergen is the largest island of the Svalbard archipelago, located approximately 76° to 81°N and 10° to 28°E (Fig. 1). The exact location and detailed information on geological settings of the study areas are listed in Tables 1 and S1. Spitsbergen is surrounded by the European Arctic Ocean, the Greenland Sea and the Barents Sea, and it is affected by three major oceanic currents; i.e., the West Spitsbergen Current (WSC), which transports relatively warm and saline Atlantic Water, and the East Spitsbergen (ESC) and Sørkapp currents (SC), which carry colder and fresher Arctic Water (Fig. 1)^55^. The fjords contain locally produced surface, intermediate and winter-cooled waters, which are influenced by the melting of glaciers and sea-ice, local precipitation and riverine runoff^11^. Whereas Wijdefjorden and Dicksonfjorden receive the WSC, Hornsund is influenced by both the warm current of the WSC and the cold current of the ESC. Furthermore, Wijdefjorden and Hornsund receive relatively large volumes of runoff from tidewater glaciers and glacifluvial rivers, whereas Dicksonfjorden only receives runoff from glacifluvial rivers^56,57^.

Surface sediment samples were collected from 35 stations located in Wijdefjorden (*n* = 6; water depths 112–322 m), Dicksonfjorden (*n* = 8; water depth 37–109 m) and Hornsund (*n* = 21; water depth 37–193 m) during three marine geological cruises on R/V Helmer Hanssen in September 2015, July 2016 and July 2017. The samples were collected using a box corer (W50 cm × H50 cm × D50 cm) (Table S1). Sub-samples were taken from the upper 1 cm of all box cores from Wijdefjorden and Dicksonfjorden, as well as from three cores from Hornsund. However, 18 sub-samples collected from the Hornsund cores during the cruise in 2015 were taken from a sediment depth range of 0–5 cm. There was no significant difference in chemical properties (THg, total organic carbon, total organic nitrogen, and δ^13^C values) between the 1 cm and 0–5 cm depth samples from Hornsund, indicating that samples of 0–5 cm depth have similar properties to the surface sediments. All subsamples were freeze-dried for two days and then ground to a powder using a mortar and pestle before further analyses.

Four bedrock samples of phyllite sandstone, calcareous shale, and black mudstone were obtained from an archive of Svalbard bedrocks that were collected during field campaigns led by the Korea Polar Research Institute from 2013 to 2015, in the Isfjorden area and central Spitsbergen. The analyzed rocks have similar compositions to the rocks surrounding our study sites (Table 1). The rock samples were also ground to powder using a ball mill.

### Analyses of THg, major and trace elements

Total Hg (THg) concentrations for surface sediments and bedrock samples were measured at the Library for Marine Samples (LIMS), KIOST and the Gwangju Institute of Science and Technology, using an automatic Hg analyzer, which undergoes thermal decomposition followed by catalytic reduction, amalgamation, desorption, and an atomic absorption module (model Hydra II Direct Hg analyzer; Teledyne Leeman Labs, Hudson, NH, USA). The THg concentrations were calibrated against marine sediment reference materials (MESS-3) for trace metals and other constituents (National Research Council of Canada). The analytical accuracies were determined to be <5%, based on replicate analyses of certified reference materials, and the analytical precision of the THg concentration measurement was determined to be <10%, based on replicate measurements of standard materials and sediment samples. To determine the concentrations of Al, Zr, Ti, Rb, Hf, Fe, Mn, Cr, V, Mo and U, the powdered samples were dissolved with a standard reference material (MAG-1) in a mixture of hydrofluoric and perchloric acids, and the elemental concentrations were measured using inductively coupled plasma atomic emission spectroscopy (Spectro Flame Modula EOP; SPECTRO Analytical Instruments Inc., Kleve, Germany) at the Korea Basic Science Institute. The analytical accuracy and precision ranged from 5 to 10%, and the concentrations were calibrated against Marine Sediment-1 reference material (certified by the U.S. Geological Survey)^58^.

### Analyses of TOC, TN, TS, δ^13^C, δ^15^N, and δ^34^S

The concentrations of total carbon (TC), total nitrogen (TN), total sulfur (TS) and total inorganic carbon (TIC) in the dried and ground sediment samples were measured using a Thermo Electron Corporation Flash EA 1112 Series NC Soil Analyzer for TC and TN, an organic elemental analyzer for TS (Vario Micro cube, Elementar, Germany), and a CO_2_ coulometer (model CM5014; UIC Inc.) for TIC (Waltham, MA, USA), at the Library for Marine Samples (LIMS), KIOST. The accuracy and precision in the analysis of these elements were <5%, based on analysis of standard reference materials (_L_-cysteine in the TC and TN analyses, sulfanilamide and acetanilide in the TS analysis, and calcium carbonate having 12% C in the TIC analysis). TOC concentrations were calculated by subtracting TIC from TC^59^.

For the carbon stable isotope analyses of OM, all sediment samples were treated with a 1 M HCl solution to remove CaCO_3_, while the δ^15^N samples were not acid-treated. The δ^13^C and δ^15^N values were measured using a CN elemental analyzer (Thermo Electron Corporation Flash EA 2000, Thermo Fisher Scientific, Germany) coupled with an isotope ratio mass spectrometer (Finnigan Delta Plus, Thermo Fisher Scientific, Germany) at the Korea Polar Research Institute^60^. The measurement error was <0.2‰ for carbon and <0.3‰ for nitrogen, and the δ^13^C and δ^15^N values were reported relative to the δ^13^C value for PDB (Pee Dee Belemnite) and air, respectively. The PDB standard and air were calibrated relative to Indiana University Acetanilide #1, USGS40, USGS41, Urea, and Thermo Soil Stand.

The sulfur (TS) contents and its isotopic value (δ^34^S) were measured with bulk sediment samples using a CNS elemental analyzer (Vario Micro cube, Elementar, Germany) coupled with a stable isotope ratio mass spectrometer (Isoprime100, Elementar, Germany) at the Korea Institute of Geoscience and Mineral Resources (KIGAM). Replicated analyses had a precision of better than 0.6%. The Vienna-Canyon Diablo Troilite (VCDT) was used for standardizing the material, and it was calibrated with NBS127 as reference.

### Calculation of the percentage for terrestrial organic carbon

Terrestrial OM is isotopically lighter (ranging from −29.3 to −25.5‰, median value = −27.3‰) than marine OM (ranging from −17 to −25‰, median value = −21‰), because C_3_ plants predominate on land over C_4_ plants at high latitudes^40,41,43,47^. In a simplified case, the use of these two endmembers allows the calculations of fractions of terrestrial or marine OM, respectively. However, the fjords on Svalbard receive not only modern terrestrial OM but also ancient OM derived from sedimentary rocks with variable δ^13^C values, depending on their origin^24^. Moreover, in regions such as the inner part of Hornsund fjord, almost the entire catchment area is covered by glaciers, cutting off any source of modern terrestrial OM^24^. The δ^13^C values for terrestrial runoff can also be affected by excrement from bird colonies, which were frequently observed in the vicinity of the Svalbard fjords. It has been reported that δ^13^C is about −26.6‰^61^. Therefore, the calculated terrestrial OM fraction must be considered as a simplified approximation.

For the calculations, we used a δ^13^C of −26.8‰ for the terrestrial OM endmember^43,47,48^, and −20.6‰ for the marine OM in the Spitsbergen fjords^43,47,48^. Using these C isotope endmember values, we calculated the relative contribution of terrestrial OM (*F*_*terr*_) to fjord sediments using the following equations:^45^

1 $$\sigma 13{C}_{org(sample)}={F}_{terr}\times \sigma 13{C}_{org(terrestrial)}+(1-{F}_{terr})\times \sigma 13{C}_{org(marine)}$$

2 $${F}_{terr}( \% )=\frac{\sigma 13{C}_{org(sample)}-\,\sigma 13{C}_{org(marine)}}{\sigma 13{C}_{org(terrestrial)}-\sigma 13{C}_{org(marine)}}\times 100( \% )$$

Where; *F*_*terr*_ (%) is the percentage of terrestrial organic carbon in the samples, δ^13^C_org(sample)_ is δ^13^C measured in the samples, and δ^13^C_org(terrestrial)_ and δ^13^C_org(marine)_ are the endmember values for terrestrial and marine OM as provided above.

### Statistical methods

The distribution of THg in Fig. 1 was created from an S100 Raster Data Set (Norwegian Polar Institute 1990) and plotted in Matlab (Version 2018). The other plots were made using a sigma plot and R software (Version 13.0; Figs. 2–4, and S1). The statistical comparison between chemical parameters and THg contents were performed using the R software.

## Supplementary information

Supplementary Information.
