# Supplementary material for: Input of terrestrial organic matter linked to deglaciation increased mercury transport to the Svalbard fjords
Source: Sci Rep. 2020 Feb 26;10:3446. doi: 10.1038/s41598-020-60261-6 (PMC7044282; doi:10.1038/s41598-020-60261-6)
Supplement: Supplementary file 1 — Supplementary Information. [file 41598_2020_60261_MOESM1_ESM.docx]

**Supplementary Information**

**Input of terrestrial organic matter linked to deglaciation increased mercury transport to the Svalbard fjords**

Haryun Kim^1^, Sae Yun Kwon^2^, Kitack Lee^2^,

Dhong-Il Lim^3^, Seung hee Han^4^, Tae-Wook Kim^5^,

Young Ji Joo^6^, Jaesoo Lim^7^, Moo-Hee Kang^8^, Seung-Il Nam^6,*^

^1^ Fundamental Research Division, National Marine Biodiversity Institute of Korea, 33662 Janghang, Korea

^2^ Division of Environmental Science and Engineering, Pohang University of Science and Technology, 37673 Pohang, Korea

^3^ South Sea Research Institute, Korea Institute of Ocean Science and Technology, 53201 Geoje, Korea

^4^ School of Earth Sciences and Environmental Engineering, Gwangju Institute of Science and Technology (GIST), 61005 Gwangju, Korea

^5^ Division of Environmental Science and Ecological Engineering, Korea University, 02841 Seoul, Korea

^6^ Division of Polar Paleoenvironment, Korea Polar Research Institute, 21990 Incheon, Korea

^7^ Geological Research Division, Korea Institute of Geosciences and Mineral Resources, 34132 Daejeon, Korea

^8^ Petroleum and Marine Research Division, Korea Institute of Geoscience and Mineral Resources, 34132 Daejeon, Korea

^*^Correspondence to: Seung-Il Nam ([sinam@kopri.re.kr](mailto:sinam@kopri.re.kr))

Division of Polar Paleoenvironment, Polar Research Institute, Incheon, 21990, Korea.

**Supplementary Information includes**

**Tables S1-S3**

**Fig. S1**

*“Scientific Reports”*

July 19^th^ 2019

**Other possibilities influencing the spatial Hg distribution in surface sediments of the Svalbard fjords**

Fe/Mn reduction under anaerobic conditions can influence the THg concentration of surface sediments^1^. When Fe/Mn oxides and hydroxide (Fe^3+^ and Mn^4+^) are reduced to Fe^2+^/Mn^2+^ under anaerobic conditions, Hg adsorbed to the reduced Fe^2+^/Mn^2+^ is released into pore water in its soluble form; therefore, it is incorporated into sediments. Low Fe/Mn reduction may, therefore, lead to higher THg contents in sediment samples, leading to positive relationships between Fe/Mn and THg contents^1^. Thus, redox proxy metals (Fe, Mn, Cr, V, Mo, U)^2^ were further analyzed to investigate the influence of redox processes on THg contents. However, we did not observe the significant correlations between Fe/Mn and THg contents in our samples (Table S3), indicating that Fe/Mn reduction had little effect on THg distribution. Additionally, as Cr and V have a very large detrital component^2^, the positive relationship between Cr/V and THg (Table S3, *p*<0.05) should be a caution to explain the redox influence on THg contents.

Coal mines had been operational in Isfjorden in western Svalbard for the last 100 years; however, the Norwegian government recently recommended the permanent closure of mining facilities at Svea and Lunckefjell close to Isfjorden (Fig. 1). A cleanup program of the surrounding areas has commenced^3^. A previous study shows that Adventfjord sediments (Fig. 1; a southern tributary of Isfjorden) have low THg concentrations, despite the presence of anthropogenic activities that can lead to elevated Hg concentrations in Spitsbergen^4,5^. Thus, it is difficult to conclude that anthropogenic Hg that is locally produced in that area is responsible for the observed THg contents in sediments elsewhere in the Svalbard fjords.

In contrast, the atmospheric transport of anthropogenic Hg probably could increase THg contents of the Svalbard fjords sediments, because Svalbard is located downwind of various Northern European countries and Russia. Numerical models have shown decreasing trends in atmospheric Hg deposition rates from southern to northern latitude in the Arctic^6^. However, a previous study found that this phenomenon is not well reflected in the Svalbard fjords because weathering of bedrock and glacier melting played essential roles in supplying THg to the Svalbard fjords^5^. Furthermore, we did not have enough information on the atmospheric Hg deposition rates at our study sites to assess the impact of atmospheric Hg input on THg distribution across the Svalbard fjord sediments.

Anthropogenic Hg transported by the Atlantic Current could potentially contribute to the spatial distribution of THg in the surface sediments of the Svalbard fjords. However, THg concentrations measured in sediments from the Barents Sea (3~5 ng g^-1^), the Beaufort Shelf (1~130 ng g^-1^) and the Greenland coast (4~280 ng g^-1^) were all lower than those observed in our fjord sediments^5^. In addition, the Hornsund sediments in our study consisted mainly of terrestrial organic matter, probably derived from local sediment sources within the fjord systems. Thus, the transport of Hg to Svalbard by the Atlantic Current seems to be a minor factor in the distribution of THg content in the fjord sediments. Furthermore, it is difficult to estimate the influence of the current on the THg contents of the Svalbard fjord sediments because quantitative information regarding the quantities of organic matter and trace metals transported from the Atlantic Ocean to Svalbard by the Atlantic Current remains limited.

**Table S1.** Locations of sampling sites and chemical properties of surface sediments from Wijdefjorden, Dicksonfjorden, and Hornsund

| Site | Lat. | Long. | THg | δ^13^C_org_ | δ^15^N | δ^34^S | TOC | TN | TS | TOC/TN |
| --- | --- | --- | --- | --- | --- | --- | --- | --- | --- | --- |
|  | (°N) | (°E) | (ng g^-1^) | (‰) | | | (%) | | |  |
| Wijdefjorden | 80.3 | 16.2 | 42.5 | –22.7 | 4.7 | 15.2 | 1.84 | 0.20 | 0.22 | 9.3 |
| (*n*=6) | 79.0 | 16.2 | 21.4 | –23.4 | 5.0 | 9.8 | 0.67 | 0.08 | 0.10 | 8.6 |
|  | 79.2 | 16.0 | 25.7 | –22.9 | 5.3 | n.d. | 0.76 | 0.09 | n.d. | 8.2 |
|  | 79.3 | 15.8 | 22.4 | –22.9 | 4.8 | 7.7 | 0.75 | 0.10 | 0.14 | 7.7 |
|  | 79.7 | 15.4 | 30.4 | –22.4 | 4.7 | n.d. | 1.36 | 0.16 | n.d. | 8.4 |
|  | 79.9 | 15.4 | 38.1 | –22.4 | 4.8 | n.d. | 1.79 | 0.21 | n.d. | 8.5 |
| Dicksonfjorden | 78.8 | 15.4 | 7.0 | –25.1 | 2.7 | 16.4 | 0.12 | 0.03 | 0.07 | 3.6 |
| (*n*=8) | 78.8 | 15.3 | 13.2 | –24.3 | 4.7 | n.d. | 0.40 | 0.06 | n.d. | 6.8 |
|  | 78.7 | 15.3 | 19.4 | –24.1 | 4.8 | 15 | 0.69 | 0.08 | 0.13 | 8.3 |
|  | 78.7 | 15.4 | 21.4 | –24.2 | 5.0 | 15.5 | 1.06 | 0.12 | 0.11 | 9.2 |
|  | 78.7 | 15.3 | 20.3 | –24.0 | 5.4 | 17.2 | 0.81 | 0.11 | 0.05 | 7.6 |
|  | 78.8 | 15.3 | 9.7 | –25.3 | 3.3 | 16 | 0.17 | 0.04 | 0.13 | 4.4 |
|  | 78.8 | 15.4 | 19.5 | –24.0 | 5.4 | 13.8 | 0.80 | 0.09 | 0.05 | 8.7 |
|  | 78.7 | 15.3 | 20.2 | –25.0 | 3.9 | n.d. | 0.63 | 0.06 | n.d. | 10.5 |
| Hornsund | 77.0 | 15.9 | 51.8 | –24.3 | 5.1 | n.d. | 1.73 | 0.17 | n.d. | 10.2 |
| (*n*=21) | 77.0 | 16.0 | 46.0 | –24.7 | 4.0 | 13.1 | 1.45 | 0.13 | 2.00 | 10.8 |
|  | 77.0 | 16.3 | 50.2 | –25.2 | 3.2 | 1.8 | 1.73 | 0.13 | 1.90 | 13.5 |
|  | 77.1 | 15.8 | 26.1 | –26.9 | 2.5 | 9.7 | 0.53 | 0.05 | 0.20 | 10.6 |
|  | 77.0 | 15.9 | 32.9 | –26.6 | 2.0 | ‒8.8 | 0.66 | 0.06 | 0.42 | 10.4 |
|  | 77.1 | 15.9 | 57.1 | –26.4 | 4.8 | 5.7 | 1.48 | 0.09 | 0.15 | 15.8 |
|  | 77.0 | 16.0 | 43.5 | –25.9 | 3.7 | 5.2 | 1.09 | 0.09 | 0.17 | 12.5 |
|  | 77.0 | 16.2 | 50.8 | –25.0 | 3.4 | ‒9.9 | 1.75 | 0.12 | 0.29 | 14.2 |
|  | 77.0 | 16.3 | 84.4 | –25.7 | 4.9 | ‒0.2 | 2.80 | 0.16 | 0.19 | 17.7 |
|  | 77.0 | 16.3 | 71.8 | –26.5 | 3.7 | 1.9 | 2.43 | 0.14 | 0.19 | 17.2 |
|  | 77.0 | 16.3 | 62.8 | –24.9 | 3.5 | ‒0.4 | 2.11 | 0.13 | 0.24 | 16.1 |
|  | 77.0 | 16.4 | 60.7 | –25.5 | 5.5 | ‒3.6 | 2.06 | 0.13 | 0.23 | 16.4 |
|  | 77.0 | 16.5 | 62.5 | –25.3 | 2.9 | ‒7 | 2.12 | 0.12 | 0.40 | 17.4 |
|  | 77.0 | 16.5 | 64.4 | –25.2 | 2.4 | ‒5.9 | 2.05 | 0.12 | 0.50 | 17.5 |
|  | 77.1 | 16.7 | 57.6 | –25.4 | 3.0 | ‒10.5 | 1.84 | 0.10 | 0.39 | 17.8 |
|  | 77.0 | 16.6 | 57.5 | –24.8 | 2.9 | ‒10.1 | 1.66 | 0.09 | 0.32 | 17.8 |
|  | 77.0 | 16.5 | 64.4 | –24.9 | 4.9 | 1.8 | 2.07 | 0.12 | 0.18 | 17.0 |
|  | 77.0 | 16.4 | 51.9 | –25.8 | 5.2 | 9.4 | 1.78 | 0.12 | 0.22 | 14.6 |
|  | 76.9 | 16.3 | 25.3 | –26.9 | 5.9 | 10.7 | 1.20 | 0.09 | 0.23 | 13.5 |
|  | 76.9 | 16.3 | 30.1 | –25.8 | 5.4 | 8.7 | 1.25 | 0.11 | 0.13 | 11.5 |
|  | 76.9 | 16.2 | 42.2 | –25.7 | 5.8 | 14.5 | 1.43 | 0.11 | 0.23 | 12.8 |

**Table S2.** Tracer metals for detrital/lithogenic and redox proxies of surface sediments from Wijdefjorden, Dicksonfjorden, and Hornsund

| Site | Lat. | Long. | Detrital/Lithogenic proxy | | | | | | Redox proxy | | | | | | |  |
| --- | --- | --- | --- | --- | --- | --- | --- | --- | --- | --- | --- | --- | --- | --- | --- | --- |
|  |  |  | Al | Ti | Zr | Rb | Th | Hf | | Fe | Mn | Cr | V | Mo | U | |
|  | (°N) | (°E) | (mg g^-1^) | (mg g^-1^) | (μg g^-1^) | (μg g^-1^) | (μg g^-1^) | (μg g^-1^) | | (mg g^-1^) | (μg g^-1^) | (μg g^-1^) | (μg g^-1^) | (μg g^-1^) | (μg g^-1^) | |
| Wijdefjorden | 80.3 | 16.2 | 58.6 | 3.9 | 182 | 100 | 9.12 | 3.67 | | 31.4 | 615 | 59.6 | 94.8 | n.d. | 2.48 | |
| (*n*=6) | 79.0 | 16.2 | 72.0 | 4.3 | 161 | 129 | 13.1 | 3.49 | | 41.2 | 1819 | 62.5 | 92.7 | 0.76 | 2.90 | |
|  | 79.2 | 16.0 | 73.9 | 4.4 | 156 | 183 | 12.5 | 3.35 | | 44 | 2751 | 64.2 | 97.5 | 2.27 | 2.77 | |
|  | 79.3 | 15.8 | 84.9 | 5.5 | 175 | 112 | 12.8 | 3.25 | | 51.4 | 757 | 78.5 | 117.2 | n.d. | 2.77 | |
|  | 79.7 | 15.4 | 68.6 | 4.9 | 237 | 116 | 11.9 | 4.37 | | 39.5 | 505 | 67.7 | 98.0 | n.d. | 2.77 | |
|  | 79.9 | 15.4 | 67.6 | 4.6 | 257 | 90 | 10.5 | 4.52 | | 35.7 | 420 | 68.8 | 100.9 | 0.16 | 2.98 | |
| Dicksonfjorden | 78.8 | 15.4 | 50.7 | 3.1 | 151 | 102 | 7.55 | 2.92 | | 26.6 | 462 | 60.4 | 86.7 | 2.61 | 4.23 | |
| (*n*=8) | 78.8 | 15.3 | 74.0 | 4.7 | 228 | 159 | 12.4 | 4.25 | | 40.5 | 536 | 67.6 | 74.8 | n.d. | 3.91 | |
|  | 78.7 | 15.3 | 80.3 | 4.8 | 201 | 168 | 12.6 | 3.88 | | 44.2 | 838 | 75.0 | 88.5 | n.d. | 4.02 | |
|  | 78.7 | 15.4 | 76.2 | 4.5 | 211 | 151 | 12.0 | 3.88 | | 42.1 | 1489 | 76.6 | 102.0 | 1.27 | 3.77 | |
|  | 78.7 | 15.3 | 78.0 | 4.5 | 182 | 160 | 11.9 | 3.28 | | 43.5 | 3088 | 78.9 | 109.7 | 4.63 | 3.69 | |
|  | 78.8 | 15.3 | 73.2 | 4.3 | 160 | 103 | 12.0 | 2.99 | | 40.8 | 2598 | 73.8 | 102.9 | 2.72 | 3.55 | |
|  | 78.8 | 15.4 | 85.5 | 4.9 | 186 | 185 | 12.5 | 3.50 | | 47.8 | 608 | 76.3 | 88.1 | n.d. | 3.56 | |
|  | 78.7 | 15.3 | 74.3 | 4.3 | 200 | 128 | 11.9 | 3.73 | | 40.5 | 1491 | 73.5 | 95.5 | 1.35 | 3.57 | |
| Hornsund | 77.0 | 15.9 | 75.5 | 4.4 | 175 | 119 | 10.5 | 3.34 | | 44.2 | 976 | 75.1 | 131.4 | 0.87 | 2.94 | |
| (*n*=21) | 77.0 | 16.0 | 88.7 | 4.9 | 191 | 132 | 11.8 | 3.87 | | 42.5 | 834 | 87.6 | 128.8 | 0.61 | 2.85 | |
|  | 77.0 | 16.3 | 91.6 | 5.4 | 229 | 130 | 12.2 | 4.17 | | 46.4 | 1235 | 90.0 | 155.7 | 1.18 | 3.17 | |
|  | 77.1 | 15.8 | 81.8 | 4.7 | 201 | 80 | 11.6 | 3.46 | | 39.1 | 468 | 95.2 | 85.2 | n.d. | 2.69 | |
|  | 77.0 | 15.9 | 83.4 | 4.6 | 186 | 142 | 14.2 | 4.18 | | 35.8 | 428 | 84.2 | 80.4 | n.d. | 3.02 | |
|  | 77.1 | 15.9 | 57.4 | 3.8 | 217 | 106 | 9.54 | 4.58 | | 26.4 | 381 | 67.5 | 86.7 | 0.66 | 3.72 | |
|  | 77.0 | 16.0 | 91.4 | 5 | 209 | 107 | 13.1 | 4.13 | | 41.5 | 492 | 91.6 | 105.9 | 0.56 | 3.28 | |
|  | 77.0 | 16.2 | 101.4 | 6.2 | 268 | 133 | 12.4 | 4.31 | | 53.1 | 968 | 101.9 | 173.5 | 1.28 | 3.53 | |
|  | 77.0 | 16.3 | 87.6 | 5.5 | 243 | 147 | 11.8 | 4.62 | | 41.2 | 501 | 99.1 | 157.5 | 2.88 | 4.60 | |
|  | 77.0 | 16.3 | 90.8 | 5.8 | 251 | 137 | 11.7 | 4.48 | | 46.1 | 742 | 95.2 | 173.0 | 2.09 | 3.88 | |
|  | 77.0 | 16.3 | 82.0 | 5.2 | 214 | 136 | 11.9 | 4.33 | | 43 | 882 | 82.7 | 154.9 | 1.91 | 3.59 | |
|  | 77.0 | 16.4 | 86.8 | 5.2 | 228 | 134 | 12.0 | 4.44 | | 44.9 | 741 | 86.1 | 164.7 | 1.13 | 3.43 | |
|  | 77.0 | 16.5 | 86.3 | 5.6 | 243 | 127 | 11.4 | 4.52 | | 46.8 | 452 | 90.6 | 178.0 | 1.55 | 3.22 | |
|  | 77.0 | 16.5 | 78.7 | 5.1 | 203 | 127 | 11.1 | 4.28 | | 41.8 | 538 | 79.5 | 158.4 | 1.22 | 3.12 | |
|  | 77.1 | 16.7 | 78.2 | 5.1 | 231 | 109 | 10.2 | 4.43 | | 40.6 | 319 | 80.8 | 159.4 | 0.59 | 2.96 | |
|  | 77.0 | 16.6 | 73.4 | 5 | 241 | 104 | 10.1 | 4.65 | | 42 | 362 | 77.9 | 135.9 | 0.25 | 3.25 | |
|  | 77.0 | 16.5 | 104.2 | 6.7 | 267 | 132 | 11.4 | 4.26 | | 55.5 | 642 | 105.1 | 201.0 | 1.47 | 3.30 | |
|  | 77.0 | 16.4 | 111.4 | 6.9 | 349 | 143 | 13.1 | 5.35 | | 55.2 | 823 | 104.0 | 171.5 | 0.88 | 4.33 | |
|  | 76.9 | 16.3 | 82.1 | 5.3 | 272 | 110 | 12.4 | 4.35 | | 42.1 | 676 | 71.9 | 96.5 | n.d. | 3.94 | |
|  | 76.9 | 16.3 | 100.6 | 5.9 | 264 | 141 | 14.8 | 4.65 | | 48.6 | 695 | 86.8 | 119.7 | 0.11 | 4.69 | |
|  | 76.9 | 16.2 | 79.7 | 4.5 | 194 | 131 | 12.7 | 4.00 | | 40.6 | 652 | 71.0 | 107.8 | 0.22 | 3.56 | |

**Table S3.** Pearson correlation coefficients and *p* values between THg, TOC, and redox proxy metals in surface sediment from Svalbard fjords.

|  |  | Fe | Mn | Cr | V | Mo | U |
| --- | --- | --- | --- | --- | --- | --- | --- |
| THg | R^2^ | 0.11 | ‒ 0.38 | 0.53 | 0.80 | ‒ 0.10 | 0.05 |
|  | *P* value | 0.54 | 0.02 | **<0.01** | **<0.01** | 0.62 | 0.78 |
| TOC | R^2^ | 0.12 | ‒0.29 | 0.46 | 0.78 | 0.01 | 0.11 |
|  | *P* value | 0.49 | 0.09 | **0.01** | **<0.01** | 0.96 | 0.52 |


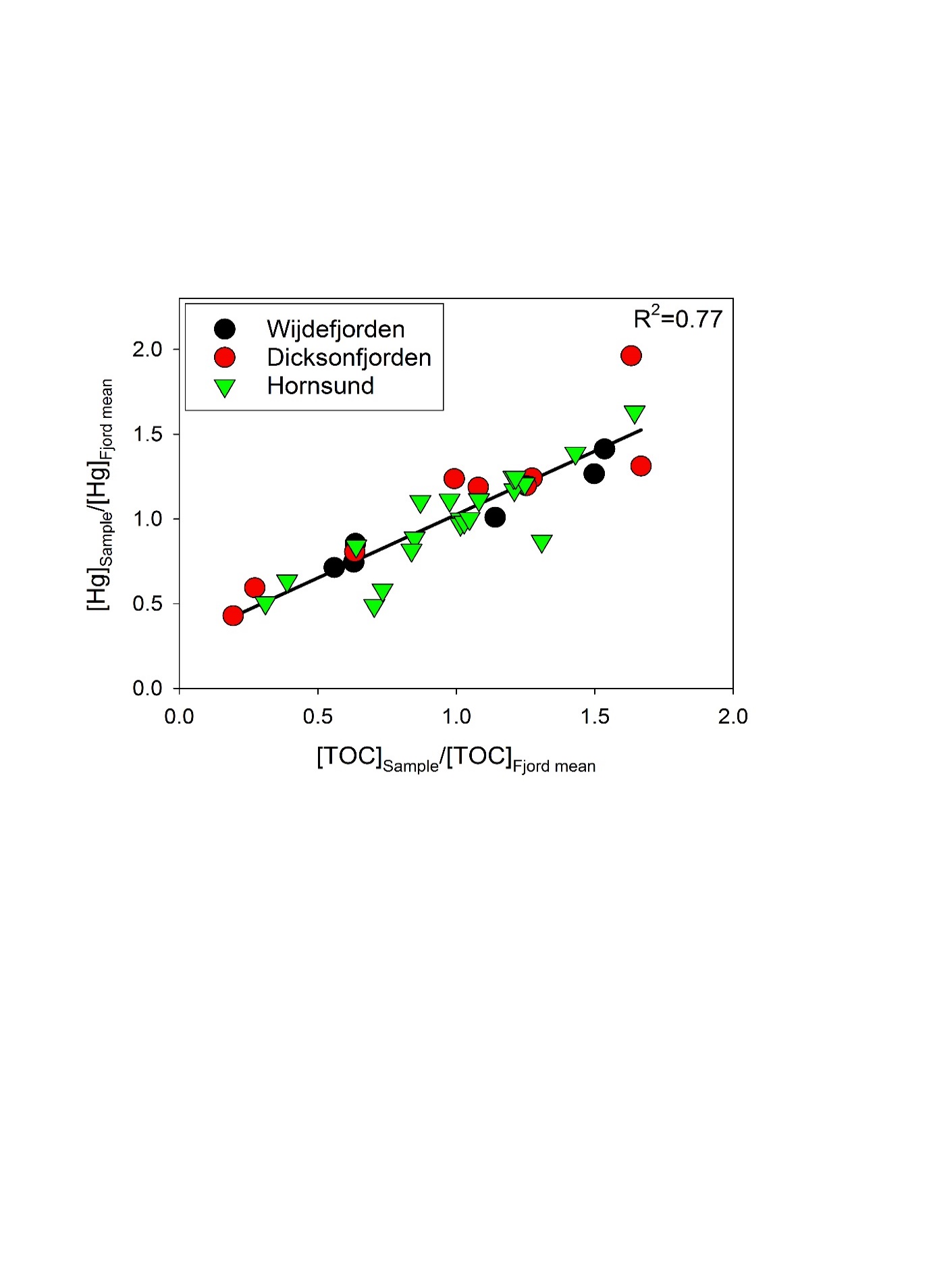


**Figure S1.** Relationship between [TOC]_Sample_/[TOC]_Fjord mean_ and [Hg]_Sample_/[Hg]_Fjord mean_ in the Svalbard fjord sediment

**Reference**

1 Gobeil, C., Macdonald, R. W. & Smith, J. N. Mercury Profiles in Sediments of the Arctic Ocean Basins. *Environ. Sci. Technol.* **33**, 4194-4198, doi:10.1021/es990471p (1999).

2 Cole, D. B., Zhang, S. & Planavsky, N. J. A new estimate of detrital redox-sensitive metal concentrations and variability in fluxes to marine sediments. *Geochim. Cosmochim. Acta* **215**, 337-353, doi:https://doi.org/10.1016/j.gca.2017.08.004 (2017).

3 Avango, D. Extracting the Future in Svalbard *in Competing Arctic Futures Palgrave Studies in the History of Science and Technology* (ed. Worms, N.) (Springer Nature, 2018).

4 Drevnick, P. E., Yang, H., Lamborg, C. H. & Rose, N. L. Net atmospheric mercury deposition to Svalbard: Estimates from lacustrine sediments. *Atmos. Environ.* **59**, 509-513, doi:10.1016/j.atmosenv.2012.05.048 (2012).

5 Bełdowski, J., Miotk, M., Zaborska, A. & Pempkowiak, J. Distribution of sedimentary mercury off Svalbard, European Arctic. *Chemosphere* **122**, 190-198, doi:10.1016/j.chemosphere.2014.11.050 (2015).
